# A Comparative Study on the Consumption Patterns of Processed Food Among Individuals With and Without Type 2 Diabetes

**DOI:** 10.3389/ijph.2025.1607931

**Published:** 2025-02-24

**Authors:** Anu Mahajan, Aditi Deshmane, Arti Muley

**Affiliations:** Department of Nutrition and Dietetics, Symbiosis School of Culinary Arts and Nutritional Sciences, Symbiosis International (Deemed) University, Pune, Maharashtra, India

**Keywords:** ultra-processed food, consumption, dietary pattern, diabetes, non-communicable diseases

## Abstract

**Objective:**

The study aims to analyse the eating patterns and consumption of ultra-processed food (UPFs) among individuals with and without diabetes.

**Methods:**

A comparative cross-sectional study was conducted across Pune, India, with 100 individuals with type 2 diabetes (T2D) and 208 without diabetes. A detailed FFQ (Food Frequency Questionnaire) developed by NOVA-UPF Screener with 33 ultra-processed food items was used to evaluate the consumption patterns of UPF.

**Results:**

Most of the participants with diabetes have a habit of eating breakfast daily (68%), prefer lunch from home (72%), and about 20% avoid eating at a restaurant. While only 45.7% of the participants without diabetes have breakfast daily, and 88.4% prefer to eat lunch outside rather than homemade food. Comparative analysis shows that all 33 UPFs were consumed significantly less by individuals with diabetes than those without diabetes (p < 0.001).

**Conclusion:**

The reduced intake of UPFs highlights greater dietary caution among individuals with T2D. Therefore, these findings emphasize the importance of promoting healthy eating habits and limiting UPF consumption among the general population to prevent the onset of metabolic conditions like diabetes.

## Introduction

The rising prevalence of non-communicable diseases (NCDs) has become a significant public health concern globally. The sharp surge is seen because of the changing lifestyle and dietary habits. The advent of technology has significantly enhanced our comfort, leading to a decrease in physical activity [1]. Not only activity but technology has also changed our eating patterns, switching us from eating whole grains and freshly produced to consuming ultra-processed food (UPFs) due to convenience, widespread availability, and enhanced palatability [2, 3]. Due to their easy availability, these UPFs have become an integral part of our daily routines, representing a significant portion of the total energy intake. For instance, sugary cereals consumed in breakfast, packaged snacks like chips and cookies consumed during the day, and ready-to-eat frozen dinners or foods ordered from outside have become everyday staples in many households. Some studies have shown that daily intake of UPFs has reached 42% in Australia and more than 56% in the UK as part of their total energy intake [4].

These foods are often high in sugars, unhealthy trans-fat, preservatives, colors, and artificial flavors, which contribute to major health issues, including obesity, cardiovascular disease, inflammatory diseases, and metabolic disorders like Diabetes [5]. The excess added sugar, unhealthy fat, and salt in UPFs gives excess empty calorie intake, which leads to insulin resistance, the root cause of most metabolic disorders [6]. It has been seen that high consumption of UPFs is associated with an elevated risk of developing Type 2 Diabetes (T2D). A meta-analysis involving a large cohort revealed that a moderate (10%) increase in UPFs leads to a 12% higher risk of developing T2D [7] and high consumption increases the risk by 31% [4]. Studies have also shown that populations consuming excess UPFs gain weight and experience metabolic syndrome, both of which are significant risk factors for diabetes [8, 9].

The rapid growth of UPFs consumption in India has increased at a compound annual growth rate (CAGR) of 13.37% from 2011–2021 and is projected to account for 39% of food retail sales by 2032 [10]. This correlates significantly with the alarming rise in diabetes and prediabetes rates, currently at 11.4% and 15.3% of the population respectively [11]. The rising prominence of these foods, particularly among urban populations, can be attributed to the evolving food environment. This landscape is increasingly dominated by food deserts and swamps, limiting access to fresh and nutritious meals. Additionally, the convenience and affordability of these foods further drive their consumption. These factors collectively promote unhealthy dietary patterns, thereby contributing to the growing prevalence of non-communicable diseases (NCDs). The high consumption and detrimental effects are also compounded by the attractive packaging and marketing technique, which often targets vulnerable populations, including children and those with lower socioeconomic status [12]. Given the rising prevalence, understanding the dietary patterns and their impact on the health is more critical than ever. This observational study compares UPFs consumption patterns among individuals with and without T2D. By analyzing these consumption patterns, we aim to identify key differences that may contribute to the increased prevalence of Type 2 Diabetes in certain groups. The findings will possibly provide valuable insights into the dietary behaviors associated of the population, thus aiming to promote healthier eating habits.

## Methods

### Study Design

This research employs a comparative cross-sectional study design to analyze the dietary patterns of individuals with and without T2D. This design allows for understanding the amount of ultra-processed food consumption at a specific time between two groups. The study was conducted in Pune, India, between April and June 2023. It was approved by the Institutional Ethics Committee (No. SIU/IEC/556). The present study follows the institute’s requirement and Helsinki’s rule. Written informed consent was obtained from all participants before data collection, ensuring they understood the study’s purpose, procedures, and any potential risks involved. Participants with T2D were recruited from five different Diabetic clinics across Pune. Individuals without diabetes were recruited from the general population and accompanied by their relatives or friends at the primary care clinic. A combination of purposive and snowball sampling methods was utilized. Purposive sampling was employed to select participants with T2D based on specific criteria, while snowball sampling helped recruit participants without T2D through referrals from initial participants.

### Inclusion and Exclusion Criteria

**FIGURE 1:**
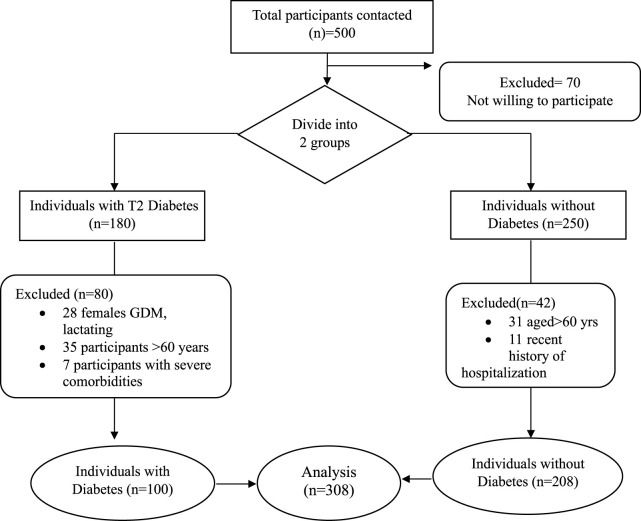
Flowchart showing recruitment, screening, and selection of participants (Pune, India, 2024).

The study includes participants from only Pune City, India, of either sex and age group 20–60 years. Confirmed T2D subjects were recruited as per their medical records or physician diagnosis with a minimum of 1 year of disease. Individuals with Type 1 Diabetes, Gestational Diabetes, pregnant women, and breastfeeding mothers were excluded from the study. Individuals with severe cognitive impairments or those unable to provide informed consent were also excluded. Nondiabetics were selected based on the absence of any known medical condition. Any major illness like cancer, HIV, or hospitalized patients were excluded from the study. The entire process of screening and enrolling is shown in Figure 1.

### Data Collection

A validated questionnaire collected demographic data, including age, gender, and health history. Anthropometric variables, like height, weight, and Body mass index (BMI), and their dietary behaviors were recorded. We carried our weighing scale and stadiometer to collect the anthropometric data, ensuring accuracy and consistency in the measurements. We validated the anthropometric variables by calibrating the equipment from time to time. We also ensured that measurements were taken by trained professionals and that standard protocols were followed. All the responses to questions were collected by conducting face-to-face interviews to ensure clarity and accuracy. This helps to understand their nutritional status, dietary preferences, and choices of eating outside food. A Food Frequency Questionnaire (FFQ) based on the NOVA-UPF Screener was utilized to evaluate ultra-processed food consumption over the last 7 days. The tool was developed by Brazil scientists and is a validated tool to quickly and easily evaluate and track the consumption of these foods [13]. The Nova classification screener was also used in a study conducted in India. The study employed the Nova classification to analyze UPF consumption patterns and their impact on nutrition in the Indian population [5] This demonstrates the utility and relevance of the Nova screener in assessing dietary patterns specific to India. Given its successful application in previous research within the Indian context, we have chosen to utilize the Nova screener in our study to ensure consistency with established methodologies and enhance the validity of our findings. Under Nova classification, foods are categorized into four groups based on extent and purpose of processing: Group 1- Unprocessed or Minimally processed; Group 2- processed Culinary Ingredients; Group 3- Processed Food; and Group 4- Ultra-processed food [3]. Ultra-processed products are mainly ready to consume and have a long shelf life, high energy, and low nutritional value [14]. The questionnaire listed 33 food items that belong to Group 4 of the NOVA classification. The food items were taken from seven categories: Bakery items, breakfast items, snacks, sauces/spreads, chocolate/candies, drinks, and sugar/sweeteners. The participants were asked to choose consumption for the last 7 days as Not consumed, Consumed Daily, or Consumed 2–3 times/week.

### Statistical Analysis

The collected data was cleaned and checked for completeness. Then was entered in MS Excel, and analyzed using SPSS v23. Descriptive statistics were used to express the demographic data in terms of mean, standard deviation, frequency (n), and percentage (%). The chi-square test was used to find the statistical significance between the groups (individuals with and without T2D). A p-value of <0.05 was considered to be significant. MANOVA (Multivariate Analysis of Variance) was performed to examine the effect of multiple items of ultra-processed food between the two groups. Wilk’s lambda, significance, and partial Eta Square Values were used to compare the effect between the groups.

## Results

### Demographics

A total of 308 participants were recruited for the study, comprising 100 individuals with T2 diabetes and 208 without T2D. The group of individuals without diabetes is double the size of those with diabetes to enhance comparative analysis. There were 60% males and 40% females with diabetes, with almost similar gender distribution among individuals without diabetes as well (62%) males and (38%) females. The mean age of the diabetic population was 46.05 ± 11.07 years. However, the nondiabetic mean age was 31.30 ± 6.26 years. The mean BMI of diabetic populations was 27.53 ± 3.67 kg/m^2,^ while of the non-diabetic population, it was 24.41 ± 3.87 kg/m^2^. Most of the diabetic population (96%) were married, whereas the individuals without T2D had fewer married people (41.9%). Both groups with 68% of the individuals with diabetes and 59.6% without T2D having completed graduation. There were 29% postgraduates in the diabetic population and 39.9% in without T2D group. Only 3% of the diabetes group and 0.5% of the without T2D group held a PhD degree. Table 1 shows the demographic characteristics of the participants.

**TABLE 1:** Demographic characteristics of the individuals with and without Type 2 Diabetes (Pune, India, 2024).

S.N.	Variables	Individuals with T2D n (%)	Individuals without T2D n (%)
1	Total Participants	100	208
2	Gender		
	Male	60 (60.0)	129 (62.0)
	Female	40 (40.0)	79 (38.0)
3	Age, years (Mean ± SD)	46.05 ± 11.0	31.3 ± 6.2
4	BMI, kg/m^2^ (Mean ± SD)	27.53 ± 3.6	24.4 ± 3.8
5	Marital Status		
	Married	96 (96.0)	87 (41.9)
	Unmarried	4 (4.0)	121 (58.1)
6	Education Status		
	Graduation	68 (68.0)	123 (59.6)
	Post Graduation	29 (29.0)	84 (39.9)
	PhD degree	3 (3.0)	1 (0.5)

### Eating Behaviors

Table 2 shows the eating behaviors of individuals with and without diabetes There was no significant difference in the dietary choices of both groups. When asked about the breakfast schedule, 68% of the diabetic participants preferred breakfast daily, while only 47.1% of the participants without T2D had it daily (p < 0.001). It was found that participants without T2D were not regular with their breakfast routine, and almost 30.2% of participants skipped their breakfast more than once a week. A statistical significance (p < 0.000) was also observed when comparing their lunch practices. Most individuals with diabetes (72%) preferred to get their lunch from home, 2% preferred eating or ordering lunch from outside, and 26% followed both practices, either getting lunch from home or eating outside food. Similarly, the comparison of UPF consumption between the individuals with and without diabetes also showed a statistical significance (p < 0.001). About 51% individuals with diabetes ate packaged food only once a week, 13% more than once a week, 26% daily, and 10% never consumed packaged food, whereas, in the case of individuals without diabetes, 15.8% ate daily, 38.9% once a week, 35.6% more than once a week and only 9.6% do not eat UPF.

**TABLE 2:** Eating behaviors among individuals with and without Type 2 Diabetes (Pune, India, 2024).

S. N	Variables	Individuals with T2D n (%)	Individuals without T2D n (%)	Chi-square (p-value)
1	Food Preferences			0.547
	Vegetarian	47 (47.0)	108 (51.9)	
	Non-Vegetarian	53 (53.0)	100 (48.1)	
2	Breakfast Schedule			
	Don’t prefer having breakfast	21 (21.0)	17 (8.17)	0.000*
	Skip breakfast once a week	5 (5.0)	30 (14.4)	
	Skip breakfast more than once a week	6 (6.0)	63 (30.2)	
	Prefer having breakfast daily	68 (68.0)	98 (47.11)	
3	Lunch Practices			
	Preferred getting lunch from home	72 (72.0)	91 (43.7)	0.000*
	Preferred eating or ordering from outside	2 (2.0)	39 (18.75)	
	Follow either	26 (26.0)	78 (37.5)	
4	Eating out at a restaurant			
	Daily	4 (4.0)	22 (10.5)	0.000*
	Once a week	63 (63.0)	154 (74.0)	
	More than once a week	13 (13.0)	77 (37.0)	
	Never	20 (20.0)	18 (8.6)	
5	UPFs consumption			
	Daily	26 (26.0)	33 (15.8)	0.000*
	Once a week	51 (51.0)	81 (38.9)	
	More than once a week	13 (13.0)	74 (35.5)	
	Never	10 (10.0)	20 (9.6)	

*p is significant at a level of <0.05.

### Ultra-Processed Food Consumption

According to the NOVA Group 4 classification, the UPF studied here was divided into seven categories: Bakery, Breakfast, Snacks, Sauces/spread, Chocolate/candies, Drinks, and Sweeteners, and the most common items used were studied in each category. Table 3 shows the complete description of UPF consumption between the two groups. Except for bread from the category of bakery items, consumption of other foods was statistically different (p < 0.0001) between the two groups. For Bread, (25%) of the subjects with diabetes and (21.2%) without diabetes did not consume bread in the last 7 days. Biscuits have emerged as popular choice among participants with diabetes as 39% consumed them daily, 33% consumed them 2–3 times per week, and only 28% did not consume them. Nearly 42.3% of subjects without diabetes did not consume biscuits, 50.9% consumed 2–3 times per week, and only 6.7% consumed daily. There was statistical significance for ready-to-eat breakfast items (p < 0.05) except for the instant oats, whose consumption was almost similar in both groups. The subjects with diabetes were seen to avoid ready-to-eat breakfast items, as 69% do not consume cornflakes, 86% avoid muesli, 96% granola, 67% instant noodles, 88% pasta, 70% instant idli- dosa mix, 91% ready-to-eat frozen meal. On the other hand, two to three times per week consumption of breakfast items was more in participants without diabetes when compared with subjects with diabetes as 38.4% ate cornflakes, 41.8% muesli, 34.1% instant oats, 41.8% granola, 72.6% instant noodles, 50% instant pasta, 49.1% instant idli dosa mix, and 44.2% frozen ready-to-eat meals.

**TABLE 3:** Ultra-processed food consumption between individuals with and without Type 2 Diabetes (Pune, India, 2024).

Food item	Individuals with T2D (n = 100)			Individuals without T2D (n = 208)			P value
	No Consumption (%)	Daily Consumption (%)	Consuming 2–3 times/week (%)	No Consumption (%)	Daily Consumption (%)	Consuming 2–3 times/week (%)	
Bakery Items							
Biscuit	28	39	33	42.3	6.7	50.9	0.000*
Bread	25	5	70	21.2	7.6	71.2	0.555
Pav/bun	65	2	33	32.7	0	66.8	0.000*
Cupcake/muffin	94	1	5	58.1	0	41.9	0.000*
Toast	54	25	21	45.1	3.4	51.5	0.000*
Khari	85	3	12	62	2.9	73.1	0.000*
Breakfast Items							
Cornflakes	69	2	29	53.4	7.7	38.4	0.033*
Muesli	86	0	14	46.2	12.0	41.8	0.000*
Instant Oats	58	2	40	61.5	4.3	34.1	0.521
Granola	96	0	4	57.7	0.5	41.8	0.000*
Instant noodles	67	1	32	27.4	0	72.6	0.000*
Instant pasta	88	0	12	49.5	0.5	50	0.000*
Instant idli/dosa mix	70	3	27	50	0.9	49.1	0.001*
Frozen, ready-to-eat meal	91	0	9	55.3	0.5	44.2	0.000*
Snacks							
Chips	71	0	29	31.7	4.32	63.9	0.000*
Namkeen/Farsaan	54	6	40	27.4	8.2	64.4	0.000*
Tortillas/Nachos	93	2	5	62.9	0.5	36.5	0.000*
Pizza/burger/wraps	69	0	31	38.9	0	61.1	0.000*
Sauces/Spreads							
Tomato Ketchup	69	0	31	44.7	1.9	53.4	0.000*
Mayonnaise	96	0	4	57.7	0	42.3	0.000*
Processed plain salted butter	63	1	36	32.2	4.8	63	0.000*
Flavored butter	96	0	4	63.9	0	36.1	0.000*
Chocolates/Candies							
Milk Chocolate	95	1	4	47.6	2.40	50	0.000*
Dark chocolates	97	1	2	47.6	0.9	51.4	0.000*
Candies	91	1	8	59.6	0.9	39.4	0.000*
Drinks							
Packaged juice	87	1	12	56.3	3.4	40.4	0.000*
Aerated Drinks	72	0	28	43.3	0.5	56.3	0.000*
Energy Drinks	94	1	5	58.7	0	41.3	0.000*
Flavored milk	96	1	3	68.7	0	41.3	0.000*
Sugar/Sweeteners							
Sugar in Tea, coffee or milk	75	16	9	16.3	33.6	50	0.000*
Sweetener in Tea, coffee or milk	91	3	6	70.2	4.3	25.5	0.000*
Flavored Yoghurt	94	0	6	54.3	1.4	44.3	0.000*
Package Kheer, Payasam	95	0	5	68.3	0	31.7	0.000*

*p is significant at a level of <0.05.

There was also statistical significance (p < 0.001) when snack consumption was compared. The consumption was seen to be less among diabetic subjects as 71% do not consume chips, 54% namkeen/farsaan, 93% tortilla/nachos, and 69% pizza/burger/wraps. A similar statistical significance (p < 0.001) was observed when sauces/spreads and chocolates/candies were compared among the two groups. Participants with diabetes were seen to avoid sauces/spreads as 69% do not eat tomato ketchup, 96% mayonnaise, 63% processed plain salted butter, and 96% avoid flavored butter. They also avoid chocolate/candies, as 95% do not consume milk chocolates, 97% dark chocolates, and 91% candies. However, participants without diabetes seemed to consume more, as 50% ate milk chocolates, 51.4% dark chocolate, and 39.4% candies two to three times per week.

A statistical significance (p < 0.001) was also observed when various drinks and sugar/sweeteners were compared. A similar trend of individuals with T2D was seen avoiding these two categories as 87% do not consume packaged juice, 72% aerated drinks, 94% energy drinks, and 96% flavored milk. They consume these drinks less often, as only 12% consume packaged juice, 28% aerated drinks, 5% energy drinks, and 3% flavored milk two to three times per week. However, the consumption was seen more in subjects without T2D, as 40.4% consume packaged juice, 56.3% aerated drinks, 41.3% energy drinks, and 41.3% flavored milk two to three times per week. Participants with T2D were also seen to avoid sugar and sweeteners, as 75% avoid sugar, and 91% avoid sweeteners in tea, coffee, or milk. They also avoided flavored yogurt, package kheer, and payasam, as 94% and 95% did not consume it over the last 7 days. However, in comparison, the consumption was more in without T2D group as 50% use sugar, 25.5% sweetener, 44.3% flavored yogurt, 31.7% package kheer, and payasam two to three times per week.

The UPF food consumption between two groups (with and without diabetes) was analyzed through multivariate tests as shown in Table 4. The multivariate test shows a statistical significance between the consumption of ultra-processed food, F (33,274) = 9.763, p = 0.000 (p < 0.05), Wilk’s Λ = 0.460, partial η^2^ = 0.540. A partial η^2^ value greater than 0.14 represents a significant effect, indicating the large proportion of the variance explained by the impact. Here, partial η2 is 0.540, indicating a large effect size. This means that 54% of the variance in ultra-processed food consumption can be attributed to the difference between individuals with and without diabetes. The large effect size implying the difference in consumption is not only statistically significant but also practically significant.

**TABLE 4:** Multivariate Analysis^a^ for consumption of ultra-processed among subjects with and without Type 2 Diabetes (Pune, India, 2024).

Effect		Value	F	Significance (p)	Partial eta square (η^2^)
Intercept	Pillai’s Trace	0.967	246.721^b^	0.000	0.967
	Wilks’ Lambda	0.033	246.721^b^	0.000	0.967
	Hotelling’s Trace	29.715	246.721^b^	0.000	0.967
	Roy’s Largest Root	29.715	246.721^b^	0.000	0.967
Individuals with diabetes vs. without diabetes	Pillai’s Trace	0.540	9.763^b^	0.000	0.540
	Wilks’ Lambda	0.460	9.763^b^	0.000	0.540
	Hotelling’s Trace	1.176	9.763^b^	0.000	0.540
	Roy’s Largest Root	1.176	9.763^b^	0.000	0.540

^a^ Design: Intercept + Diabetic Status.

^b^ Exact statistic.

Test-of-between subjects show the significant consumption of different ultra-processed food among the both groups and a statistical significance was observed among consumption of pav/bun, cupcake/muffin, toast, khari, muesli, granola, instant noodle, instant pasta, instant idli dosa mix, frozen ready product, chips, tortilla/nachos, fried namkeen, pizza/burger/wraps, Milk chocolate, Dark chocolate, Candies, tomato ketchup, mayonnaise, butter (plain or flavored), package juice, cold drinks, energy drinks, flavored milk, sugar, sweetener, Flavored yogurt, package kheer or payasam. A significant large effect is seen in certain food items as partial η^2^ value is found to be greater than 0.14, like Granola (0.153), Instant noodles (0.140), milk chocolate (0.212), dark chocolate (0.235), Mayonnaise (0.154), sugar in tea, coffee, milk (0.302) and flavored fruit yogurt (0.154) as shown in Table 5.

**TABLE 5:** Tests of Between-Subjects Effects of ultra processed food consumption among individuals with and without Type 2 Diabetes (Pune, India, 2024).

Category	Dependent variable	Mean square	F	Significance	Partial Eta Sq
Individuals with T2D vs. without T2D	Biscuit	0.090	0.109	0.742	0.000
	Bread	0.169	0.241	0.624	0.001
	Pav/bun	28.685	31.817	0.000*	0.094
	Cupcake/muffin	35.648	49.084	0.000*	0.138
	Toast	10.404	12.045	0.001*	0.038
	Khari	14.338	19.023	0.000*	0.059
	Cornflakes	10.292	2.827	0.094	0.009
	Muesli	30.927	41.010	0.000*	0.118
	Instant oats	0.660	0.720	0.397	0.002
	Granola	39.145	55.168	0.000*	0.153
	Instant noodle	42.394	49.883	0.000*	0.140
	Instant pasta	39.502	48.499	0.000*	0.137
	Chips/Fries	37.193	43.810	0.000*	0.125
	Nachos/Tortillas	25.590	36.761	0.000*	0.107
	Fried Namkeen/Farsan	17.578	21.133	0.000*	0.065
	Pizza/Burger/Wraps	24.405	26.353	0.000*	0.079
	Milk Chocolates	58.917	82.296	0.000*	0.212
	Dark Chocolates	65.983	94.155	0.000*	0.235
	Candies	26.640	35.812	0.000*	0.105
	Tomato Ketchup/Sauce	14.699	15.618	0.000*	0.049
	Mayonnaise	39.641	55.532	0.000*	0.154
	Salted Processed Butter	22.538	25.541	0.000*	0.077
	Packaged Flavored Butter	27.761	41.001	0.000*	0.118
	Packed Juice	23.615	30.297	0.000*	0.090
	Aerated cold soft drinks	21.926	23.614	0.000*	0.072
	Energy Drinks	34.710	47.939	0.000*	0.135
	Flavored Milk	17.026	30.392	0.000*	0.090
	Sugar in Tea, Coffee, Milk	67.066	132.501	0.000*	0.302
	Sweetener in Tea Coffee Milk	10.962	18.413	0.000*	0.057
	Flavoured Fruit Yogurt	40.986	55.632	0.000*	0.154
	Packaged Kheer Payasam	19.302	29.646	0.000*	0.088
	Instant Mix Idli Dosa	11.935	12.837	0.000*	0.040
	Packaged Branded Ready to Eat Frozen	33.988	43.843	0.000*	0.125

*p is significant at a level of <0.05.

## Discussion

The present study examined the consumption patterns of UPFs among individuals with and without T2D. There was a significant difference in eating habits, food preferences, and consumption of UPFs between the two groups. It was seen that individuals with T2Dare more aware of their health conditions and have breakfast daily, prefer to have lunch from home, avoid eating outside, and package food to maintain their blood sugar levels. However, existing literature says that prior studies on T2D participants suggested showcase that they do not have healthy eating habits [15, 16]. Diabetes is a metabolic disorder and can be managed by proper awareness of eating habits. A study was done to educate people about the benefits of the Mediterranean diet, which has helped control sugar levels and improve lipid profile and weight loss in 3 months [17]. Our study observed that most participants reported having breakfast daily; however, we did not assess how this behavior impacts diabetes risk. Future research could explore this potential relationship. In our study, participants seemed aware of their eating habits, as many of them had breakfast daily. There are associations between breakfast consumption and a lower risk of Diabetes Conversely, the population without T2D tends to eat more packaged food and prefers to eat more at restaurants or outside food. A recent study also shows similar trends among the urban Indian middle-class populations, highlighting that consumers in India are opting for more processed food due to various factors such as globalization, urbanization, and changing socio-cultural dynamics. The food choices are driven by convenience, availability, and marketing of processed food [18].

The observed healthier eating patterns among individuals with diabetes may be attributed to prior dietary counseling received at diagnosis. Dietary and lifestyle interventions are often recommended as first-line management strategies for diabetes, emphasizing the reduction of ultra-processed food intake and promoting whole, nutrient-dense food [19]. Several studies have shown that structured nutritional education programs can significantly improve dietary behaviors, leading to better glycemic control and weight management [7, 8]. In our study, individuals with diabetes seemed aware of their eating habits and consuming homemade meals. However, we did not collect information on whether participants with T2D had received dietary counseling at the time of diagnosis. Since nutritional education plays an important role in diabetes management by influencing food choices and dietary habits, the absence of this data may introduce an unaccounted variable, potentially affecting the study behavior. Further research should include this variable to understand its impact on dietary behavior better.

The present study analyzed the different UPFs based on Nova classification. We observed an increasing trend of bakery items like bread and biscuits, which have become integral to every household. Here, marketing influences consumer choices and dietary habits [20]. Brands often position their products as “Diabetic-friendly” by highlighting specific health benefits like sugar-free, whole grain, and high fiber. We did not directly evaluate the influence of marketing or advertisement on dietary choices. However, previous research studies suggest that marketing may play a role in promoting ultra-processed foods as convenient or healthful options, warranting further investigation. Attractive packaging can also enhance the perceived value of processed food. It has been found that consumers rely on visual cues and branding rather than nutritional information when making food choices, leading to increased consumption of processed food [21]. A study sought to explore the main stakeholders, frameworks, motivations, and interactions within the global UPF system that have contributed to the widespread prevalence of UPFs in population diets. According to this study, the systems thinking approach underscores that diminishing UPF consumption necessitates tackling interrelated factors like as food cost, cultural changes, and marketing strategies [22]. By acknowledging these linkages, our work introduces a population-specific viewpoint, so underscoring the necessity for comprehensive solutions.

Ultra-processed snacks are engineered to be highly palatable, exploiting human cravings for sweetness and saltiness. The manipulation of flavors makes them more appealing, leading to increased consumption [23]. This can be seen in the case of the consumption of snacks in the study, where both groups show consumption of chips, namkeen, and nachos, pizza, burgers due to their taste, convenience, and affordability. The combination of sugar, salt, and fat in ultra-processed food can create addictive eating behaviors. Various studies show that these foods trigger reward pathways in the brain, a similar behavior to addictive substances, and can lead to overconsumption and increase the risk of disease [24, 25]. The consumption of sugar-sweetened beverages was also higher in nondiabetic participants than in the diabetic group. Regular intake of these beverages is linked to insulin resistance and fatty liver [26]. A systematic review highlighted that a higher intake of sugar-sweetened beverages correlates to a 13% increased risk of T2D and an 8% increased risk of cardiovascular diseases per additional consumed daily serving [27].

The higher consumption of ultra-processed food among individuals without T2D raises significant public health concerns. Regular intake of these foods is associated with an increased risk of developing non-communicable diseases, including T2D, obesity, and cardiovascular diseases. The increased consumption of ultra-processed food may add fuel to this fire. Not only NCDs, high intake of such food can even lead to mental disorders like a 48%–53% increased risk of anxiety and a 22% increased risk of depression [28]. The potential reasons why individuals with T2D may consume less ultra-processed food is that they are more likely to engage in health-conscious behavior because of the dietary recommendations given by dietitians, like avoiding foods high in sugar, unhealthy fats, and additives that are prominent in ultra-processed food. The second reason can be influenced by behavioral factors like increased health literacy and motivation to manage the disease. Overall, this research emphasizes the critical role of targeted nutritional education and supportive policymaking in reducing the public health burden associated with UPF consumption.

### Limitations

The current study has certain limitations that have to be acknowledged. The present study uses the Nova classification system, which has notable limitations. While the Nova framework effectively categorizes foods based on the extent of processing, it does not assess their nutritional content. This means that nutritionally balanced foods may be included in Nova Group 4 if they have undergone extensive industrial processing. For instance, dark chocolate, classified as ultra-processed (Nova group 4), contains flavonoids with positive health outcomes, such as improved cardiometabolic markers and reduced inflammation, However, plain yogurt, which is rich in protein, calcium, and probiotics, is minimally processed (Nova Group 1) does not fall under the ultra-processed category. Misinterpretations of the Nova classification may lead to overestimations of unhealthy food intake in the study population. Additionally, reverse bias presents a significant limitation. Participants with T2D may underreport their consumption of UPFs due to social desirability or perceived stigma, potentially leading to skewed data. This could result in an underestimation of actual UPF consumption within this group. A relatively small sample size, participants were from one city and may not represent the entire nation’s population. The study relied on self-reported data for dietary and anthropometry information, subject to recall bias. Participants may underreport or overreport the consumption of particular food, particularly unhealthy or socially undesirable items. The present study is a cross-sectional study and cannot establish causality between the consumption of processed food and the development of NCDs. A longitudinal study design would be more appropriate for examining the long-term effects of dietary habits on increased risk.

### Conclusion

The study compared the consumption of UPFs among two distinct populations. The results highlighted that the T2D population is more aware and consumes less processed food than those without T2D. One of the reasons would be restrictions due to medical conditions, and information physicians share about healthy eating. This also suggests a potential lack of awareness among the healthy group regarding the health risks of consuming UPFs regularly. The finding highlights the need for nutritional education among the non-diabetic population to promote healthier eating habits and reduce reliance on ultra-processed food. As the prevalence of undiagnosed diabetes and pre-diabetics is on the rise, especially among the Indian population, there is an urgent need for awareness sessions. Further research can explore the specific factors contributing to differences in consumption patterns between the two groups and provide insights that inform public health strategies and help promote healthier eating habits across the broader population.
